# mvp – an open‐source preprocessor for cleaning duplicate records and missing values in mass spectrometry data

**DOI:** 10.1002/2211-5463.12247

**Published:** 2017-06-19

**Authors:** Geunho Lee, Hyun Beom Lee, Byung Hwa Jung, Hojung Nam

**Affiliations:** ^1^ School of Electrical Engineering and Computer ScienceGwangju Institute of Science and Technology (GIST)Korea; ^2^ Molecular Recognition Research CenterKorea Institute of Science and Technology (KIST)SeoulKorea

**Keywords:** dirty data, duplicate record, mass spectrometry, missing value, MS data preprocessor, R package

## Abstract

Mass spectrometry (MS) data are used to analyze biological phenomena based on chemical species. However, these data often contain unexpected duplicate records and missing values due to technical or biological factors. These ‘dirty data’ problems increase the difficulty of performing MS analyses because they lead to performance degradation when statistical or machine‐learning tests are applied to the data. Thus, we have developed missing values preprocessor (mvp), an open‐source software for preprocessing data that might include duplicate records and missing values. mvp uses the property of MS data in which identical chemical species present the same or similar values for key identifiers, such as the mass‐to‐charge ratio and intensity signal, and forms cliques via graph theory to process dirty data. We evaluated the validity of the mvp process via quantitative and qualitative analyses and compared the results from a statistical test that analyzed the original and mvp‐applied data. This analysis showed that using mvp reduces problems associated with duplicate records and missing values. We also examined the effects of using unprocessed data in statistical tests and examined the improved statistical test results obtained with data preprocessed using mvp.

**Abbreviations**
AMI: acute myocardial infarction
*m/z*: mass‐to‐charge ratio
MI: multiple imputation
MS: mass spectrometry
mvp: missing values preprocessor
ToF: time‐of‐flight

Mass spectrometry (MS) data are widely used to analyze various biological phenomena by producing mass spectra patterns for the associated chemical or biological species, such as compounds, metabolites, peptides, and proteins. Analyses of MS data consist of ionization analyses, mass analyses, and chemical species detection 1. MS measures the mass‐to‐charge ratio (*m/z*) of an ionized chemical species, and when MS is coupled to a second MS, known as tandem MS or MS/MS, it allows the detection of the fragment ions of a selected ion that is an identifier for a molecule. Interpreting MS results enables researchers to biologically interpret many domains. MS data are widely used in proteomics, metabolomics, and drug development 2, 3, 4. For example, researchers can utilize MS to analyze complex protein mixtures, identify metabolites, or discover target drugs. Over time, the performance of MS systems has continually improved, which has allowed for the analysis of as‐yet unanalyzed chemical species. Many types of MS systems have been developed, including time‐of‐flight (ToF) and Orbitrap spectrometers 5, 6. Each mass analyzer has different operating principles and a different mass resolution. In this study, we focused on data generated from an LC‐ToF/MS machine and used these data to verify the performance of our open‐source software.

Unintended duplicate records and missing values are representative dirty data problems associated with MS data and should be mitigated to improve the data analysis. The process of handling dirty data is also called data cleansing, and it has been performed in statistics and data science, when dealing with large numbers of records, tabular data, or databases. We often encounter duplicate records that originate from identical molecules and missing values in the measured intensity signals in MS data sets. These problems frequently occur for technical or biological reasons 7. These dirty data problems can affect the power of statistical and machine‐learning tests 8. Therefore, methods have been developed to alleviate these problems. Duplicate detection is generally identified faster if the data are sorted by a key. We used this property to detect candidate duplicate records in our research. Compared with duplicate detection, many approaches have been developed to handle missing values. Simple methods of handling missing values include filling them in with ‘0's or imputing the missing data point with the mean value. However, these simple methods can produce biased values and results 9, 10. The k‐nearest neighbor (k‐NN) approach is a popular imputation method with good performance 7, 8. However, k‐NN has a disadvantage in that its performance depends on the number of complete records. Another common approach for efficiently imputing missing values is the multiple imputation (MI) method. MI is an efficient method, and its performance has been verified in several studies 11, 12. Recently, machine‐learning methods, including naïve Bayesian‐, neural network‐, and decision tree‐based imputation methods, have also been widely used in various domains 13, 14, 15. However, these methods focus only on imputing missing values. With tabular data, which is the most common structured data form for data analysis, dirty data problems might include both unintended duplicate records and missing values. The methods described above are not dedicated methods for MS data and thus cannot effectively handle unintended duplicate records. Additionally, the previously discussed missing value imputation methods rely on statistics or mathematics without considering the basic domain properties of the MS data.

To properly handle problems of dirty data in MS data, methods are required that can preprocess duplicate records and missing values while also considering the MS data characteristics. We considered the key identifier in MS data as the implementation core for the missing values preprocessor (mvp) open‐source platform. Key identifiers of chemical species are observed in MS data, such as the *m/z*, retention time, and intensity. mvp uses the property of MS data wherein identical chemical species have the same or similar values for key identifiers. In other words, similar *m/z* values will be accompanied by similar intensities, and this property can be used to determine whether or not chemical species are identical 16. Based on this idea, mvp can detect and process duplicate records by examining the similar values of key identifiers for individual chemical species. mvp merges duplicate records into one record because it considers each duplicate record as an identical chemical species. Thus, in the merging process, certain intensity columns that have missing values can be imputed if the complete values of other records are available. Figure 1 shows the overall mvp process and illustrates the previously explained chemical species properties. The processing flow of mvp includes the following four separate steps: (a) extracting duplicate candidate records by using *m/z* and the retention time, (b) calculating the pairwise record similarity of the intensities in the candidate group, (c) converting record information to a graph structure, and (d) merging similar records into one record. Detailed descriptions of each step are provided in the Materials and methods section.

**Figure 1:**
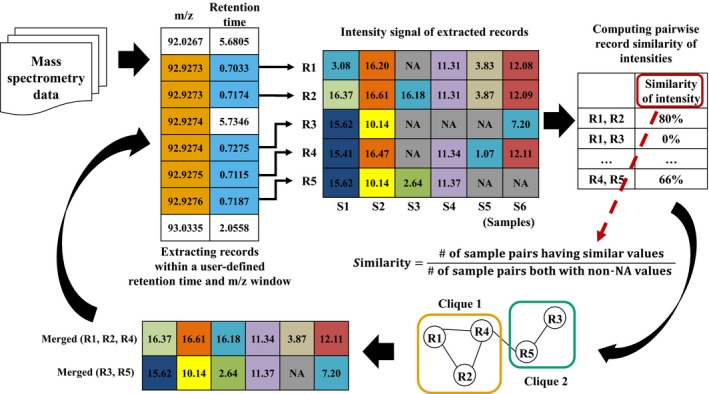
Overview of mvp processing. The mvp procedures include extraction and merging steps. Using MS data identifiers, mvp extracts identical record candidates. After calculating the pairwise similarity of all candidates, mvp constructs a graph and identifies cliques. The clique record components are merged by mvp using user‐specified parameters.

## Materials and methods

### Materials

#### Sample collection

The study subjects were sorted into the following four groups: (a) control (*n* = 40), (b) acute myocardial infarction (AMI, *n* = 42), (c) angina pectoris (*n* = 72), and (d) unstable angina (*n* = 23). Serum samples were obtained by placing blood samples collected from the antecubital vein of the forearm into serum‐separating tubes and centrifuging for 10 min at 956 ***g***.

#### Sample preparation

For protein precipitation, an ice‐cold methanol/water (3 : 1) solution was added to the serum and centrifuged at 20 817 ***g*** for 15 min at 4 °C. The supernatant was diluted with distilled water to half the volume of the supernatant, and it was then injected in the ultra‐performance liquid chromatography (UPLC) (ACQUITY^®^; Waters, Milford, MA, USA) coupled with a QToF‐MS (SYNAPT™ G2; Waters).

#### Data collection

All samples were analyzed in a randomized order in both positive and negative ionization modes. The raw MS data were obtained by masslynx software version 4.1 and markerlynx software (Waters) after deconvolution, which included peak detection, alignment, and normalization.

### Development process

#### Description of overall process and user‐specified parameters of mvp

mvp provides various user‐specified variables. First, mvp supports different window sizes for *m/z* and the retention time. In Fig. 1, because the window size of the retention time is 0.02, only the five records colored in blue that satisfy this condition were extracted. Using the data in Fig. 1, mvp first extracts the largest clique composed of R1, R2, and R4. After extracting the candidate records using *m/z* and the retention time, mvp calculates the possible pairwise similarity of the record intensity. The pairwise similarity of the record intensity is calculated as the number of samples having similar intensity values divided by the number of samples having both non‐NA values. In mvp, the definition of similarity of intensity is when the difference is within ±5%. For example, if the intensity value of record 1 is 100, and record 2 has an intensity between 95 and 105, they are judged to have similar values. In Fig. 1, R1 and R2 have four similar intensity values (S2, S4, S5, and S6) and five complete intensity values (S1, S2, S4, S5, and S6). Thus, the similarity of R1 and R2 is 4/5. The similarity of R4 and R5 can be computed in the same way, with two similar intensity values (S1 and S4) and three complete intensity values (S1, S2, and S4), yielding 2/3. The merging process continues for the extracted cliques. mvp uses one method, such as the maximum, median, mean, or minimum, when merging the component records in the clique to one record, and this method can be specified by the user. For example, when the maximum method is applied, the value of S1 in clique 1 is 15.41 because 15.41 is the maximum value among 3.08, 10.53, and 15.41. Similarly, S3 in clique 1 is 16.18 because 16.18 is the maximum value excluding NA. From the calculation of S3 in clique 1, we can identify how mvp interpolates the data for the missing value problem.

#### Duplicate candidate detection by identifiers (*m/z*, retention time)

In the first step, to extract duplicate candidate records, mvp rearranges the input tabular data using a key identifier of the chemical species, such as the *m/z* or retention time. Table 1 shows an example of an input format of the MS tabular data. Users can set indices of key identifiers before executing mvp. For example, users can set column indices for *m/z* and retention time or set one column index based on the sorting key. Users can also assign criteria for similar key identifiers. In this step, mvp filters out a candidate group of duplicate records. The top left of Fig. 1 shows five duplicate candidate records that were extracted when the similarity criteria for *m/z* and retention time were 0.002 and 0.3, respectively.

**Table 1:** General tabular form of MS data (*m/z*, mass‐to‐charge ratio; RT, retention time)

Compound identifier	*m/z*	RT	Intensity of sample_1_	…	Intensity of sample_*n*_
Compound_1_	92.9273	0.7033	11.31	…	12.08
Compound_2_	92.9274	0.7174	11.34	…	NA
…	…	…	…	…	…

#### Computation of pairwise record similarity of intensity

After extracting the duplicate candidate records, mvp calculates the pairwise record similarity of the intensity in the candidate group. When constructing the graph structure, these calculated pairwise record similarities and the record similarity threshold, which can be specified by the user, are used to connect the edges. The record similarity value is defined as the number of samples with similar values divided by the number of samples with no unavailable (NA) values. For example, R1 and R2 in Fig. 1 have 80% similarity because there are five samples that have no NA values (S1, S2, S4, S5, and S6) and four samples that have similar values (S2, S4, S5, and S6).

#### Constructing the graph form and finding possible cliques

mvp builds the graph structure based on the results from step 2. mvp considers each record in the duplicate candidate group as a vertex and makes edges that have larger pairwise similarities than the record similarity threshold specified by the user. mvp identifies all possible cliques in the graph after constructing the base graph. The right bottom of Fig. 1 shows the base graph structure when the record similarity threshold is 50%.

#### Merging duplicate records and imputing missing values

Finally, mvp conducts a merging procedure with regard to the constructed graph structure. mvp extracts the clique in an order from large to small. When extracting the cliques, mvp checks whether the current clique is independent, which indicates whether the component of cliques overlaps with the previously selected cliques. A detailed example is shown in the Appendix S1.

### Simulation data construction

To validate the performance of mvp in terms of qualitative analyses, we generated simulation data based on heart disease LC/ToF‐MS data as explained in the material section. We generated simulation data that are similar to actual MS data, which contain duplicate records and missing values. The simulation data were generated by randomly increasing the number of records, inserting random noise into the intensity values, and randomly incorporating missing values. Performance measurements were used to compare the answer data and the results after applying mvp to the simulation data. We measured how well mvp restored the simulation data. We performed 30 repetitions and calculated the standard error to determine the reliability of the experiment.

### Comparison of the statistical test performance before and after applying mvp

Additional experiments were conducted to ascertain whether the duplicate records and missing values affected the statistical testing. We assumed that preprocessing these dirty data problems would improve the performance of the statistical or machine‐learning tests. For example, we evaluated whether the accuracy of classification could be improved or the number of significant metabolites can be increased when applying Student's *t*‐test. These evaluations used LC/ToF‐MS heart disease data, which represented positive and negative ion mode data with preprocessing.

## Results

### Quantitative analysis by interpreting the changes in duplicate records and missing value proportions

We assessed the performance of the mvp software (Computational Systems Biology Lab., School of Electrical Engineering and Computer Science (EECS), Gwangju Institute of Science and Technology (GIST), Gwangju, Korea) via quantitative and qualitative analyses. We obtained preprocessed data that contained missing values and duplicate records via mvp for the quantitative analysis. The number of duplicate records was reduced after applying mvp (Table 2). Table 2 shows the number of original data records and the mvp‐applied data specified by the different record similarity parameter thresholds. If the record similarity parameter threshold was assigned a small value, too many records could be eliminated, suggesting that the output may contain mistakenly merged records that are not duplicate records. Conversely, if this parameter was set to a large value, users can obtain more robust results but a relatively small record reduction advantage.

**Table 2:** Record reduction rates before and after applying mvp with different record similarity variable thresholds

	Original data	mvp‐processed data (50% similarity)	mvp‐processed data (70% similarity)
Positive ion mode	3908	3360 (−14.0%)	3567 (−8.7%)
Negative ion mode	18 253	13 874 (−24.0%)	15 648 (−14.3%)

**Figure 2:**
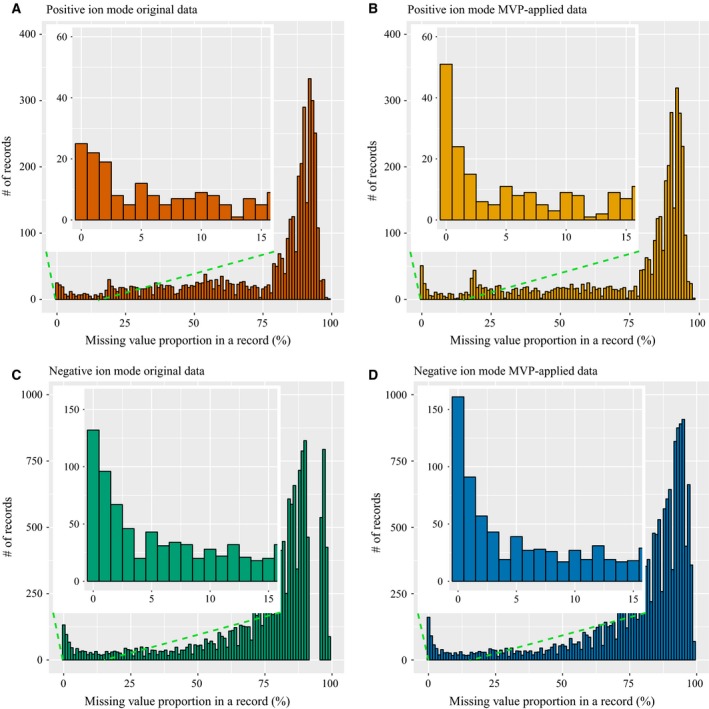
Histogram for missing value problems before and after applying mvp. (A), (B) and (C), (D) Heart disease positive and negative ion mode results, respectively. (A) and (C) Original results. (B) and (D) mvp‐processed results obtained using the following default parameter values: (a) *m/z* similarity threshold of 0.001, (b) retention time similarity threshold of 0.3, and (c) record similarity threshold of 70%.

Managing missing values is another benefit of mvp as shown in Fig. 2, which is formed by four histograms from the same data already described in Table 2. The *x*‐axis reflects the missing value proportion in a record, and the *y*‐axis represents the number of total records corresponding to the missing value proportion. Figure 2A and C shows that the original data have a large number of incomplete records, with the proportion on the *x*‐axis greater than approximately 90%, and few complete records, with the proportion on the *x*‐axis at 0% for the two different data sets. Figure 2B,D represents the missing value proportion after applying mvp, and the number of complete records increases from Fig. 2B,D to Fig. 2A,C. This result indicates that mvp properly manages missing values.

### Accuracy assessment by qualitative test

**Figure 3:**
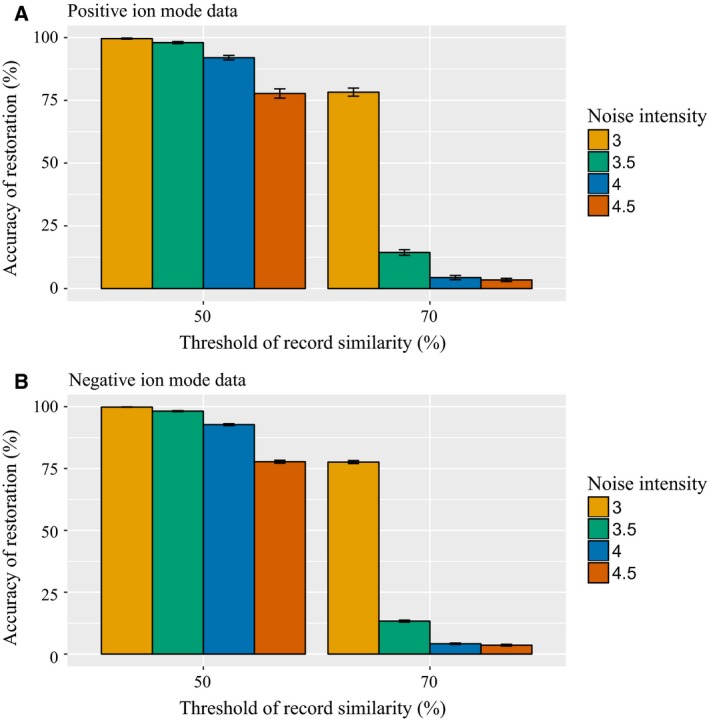
mvp performance accuracy for simulation data record restoration. (A) and (B) Results based on positive and negative ion mode data for heart disease, respectively. Each simulation was tested by assigning a different record similarity and noise intensity threshold. The error bars illustrate the mean ± SEs.

In addition to the quantitative analysis, we also conducted a qualitative analysis via simulation testing. To obtain the simulation data, we removed all records with NA values. From the NA‐removed data, we created simulation data similar to the actual MS data, which contain duplicate records and missing values. The simulation data were generated by randomly increasing the number of records, inserting random noise to the intensity values, and randomly incorporating missing values. When inserting the random noise to the intensity values, we add the value of the normal distribution with four noise intensities, which were assigned based on the standard deviation value of the normal distribution. Figure 3 suggests how well mvp recovers simulation data that have deliberately produced noise. We applied a different record similarity threshold for the two different data sets. The *x*‐axis of Fig. 3 represents the parameter value record similarity threshold, and the *y*‐axis represents the restoration accuracy calculated by comparing the original data, which are the source of the simulation data, and the mvp‐applied data. Each experiment was conducted 30 times, and the accuracy of restoration and standard error of the mean were recorded. We found that higher noise intensity corresponded to a lower accuracy of the restoration. Moreover, the strict record similarity threshold of 70% allowed the mvp process to recover less data compared with when applying a threshold of 50%.

### Statistical test results before and after applying mvp

**Figure 4:**
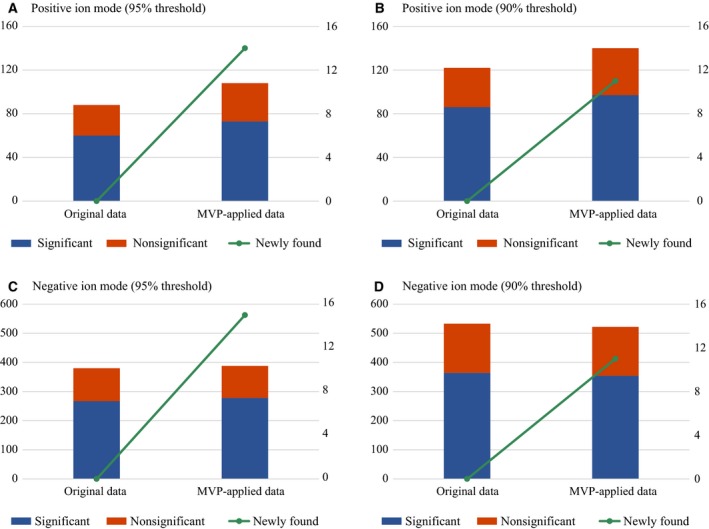
Results from FDR test (Benjamini–Hochberg procedure) after Student's *t*‐test before and after the application of mvp. (A), (B) and (C), (D) Results based on positive and negative ion mode data for heart disease, respectively. The height of the red box illustrates the proportion of chemical compounds with FDR < 0.05, and the sum of the heights of the blue and red boxes illustrates the total number of chemical compounds. The green line with the secondary *y*‐axis shows the number of significant chemical species that were newly identified after applying mvp.

Lastly, the numbers of significant features after applying mvp were analyzed. We examined the data sets by selecting records that had proportions of complete values greater than 95% and 90%. Finally, the following four data sets were obtained: (a) positive ion mode with a 95% threshold, (b) positive ion mode with a 90% threshold, (c) negative ion mode with a 95% threshold, and (d) negative ion mode with a 90% threshold. From each data set, we generated original data and mvp‐applied data. The original data were generated by applying a k‐NN imputation to the previously generated data. mvp‐applied data were generated by applying mvp first and then a k‐NN imputation to the previously generated data. We then applied Student's *t*‐test to the original and mvp‐applied data and compared the results for the control and AMI groups. The *P*‐value of each metabolite was calculated after applying Student's *t*‐test. The FDR test (Benjamini–Hochberg procedure) was also applied to compensate for the *P*‐values 17 and determine the number of significant (FDR < 0.05) metabolites. Furthermore, we also calculated the number of significant metabolites that were not identified in the original data because of duplicate records and missing values but were newly identified after preprocessing with mvp. Figure 4A–D illustrates the results of four independent experiments and indicates that significant metabolites that were not previously discovered were identified after applying mvp preprocessing.

## Discussion

MS data present dirty data problems that include duplicate records and missing values, and these problems may cause the degradation of statistical tests or machine‐learning algorithms. Thus, we implemented an R language‐based open‐source software named mvp to preprocess duplicate records and missing values in MS data. mvp uses the property of MS data wherein identical chemical species also present the same values for key identifiers, such as the *m/z*, intensity, and retention time.

We verified the performance of mvp via quantitative and qualitative approaches. For MS data, duplicate records and missing values were managed by mvp preprocessing. Furthermore, a comparison of the original and mvp‐applied data indicated that dirty data problems could actually lead to decreased statistical test performance. These results were consistent with our assumption regarding the relationship between dirty data problems and statistical test performance degradation. In this regard, researchers who work with MS data can use mvp with various user‐specified parameters to preprocess MS data before applying these data to statistical tests or machine learning.

Certain minor limitations were observed in the application of mvp because of the data types and algorithms used in this study. First, many types of MS equipment are available, including ToF/MS and Orbitrap spectrometers. Only the results for the LC/ToF‐MS spectrometer are described in the main text, although we also experimented with Orbitrap data and found that mvp produced good results. An examination of dirty data in the Orbitrap data yielded different result trends compared with the ToF‐MS data because the two machines have different characteristics. Another problem involves the identification of cliques in a dense graph. Finding cliques is a well‐known NP‐complete problem 18, 19. Thus, if the graph is too dense, then finding the cliques will take a long time. To handle this problem, mvp automatically changes the algorithm based on finding cliques. For a dense graph, mvp finds a pair of records with the best similarity and merges them, and changing the algorithm can reduce the computation time.

The main objective of mvp is merging multiple records that potentially originate from a single substance. mvp was designed to set a threshold of record similarity to fit the researcher's objective. For example, if a researcher wants to minimize false positives, the threshold of record similarity can be set to a high value such as 0.9 or 0.95, while if the researcher needs to obtain more candidates even at the risk of collecting more false positives, it is possible to set the threshold of record similarity to a low value such as 0.3 or 0.5.

When using mvp to analyze MS data, researchers need to take a careful approach depending on the resource type such as a metabolite or protein. Unlike metabolites, in the case of proteins, different peptides could have similar identifiers (*m/z*, retention time). Therefore, the researchers should consider the characteristics of the resource types when setting the user‐specified parameters provided by mvp.

Our findings indicate that the open‐source software mvp can facilitate the preprocessing of MS data with respect to duplicate records and missing values. The various validation procedures showed that mvp properly manages duplicate records and missing values. Moreover, the performance of statistical tests was improved by the application of mvp because new significant metabolites were identified after preprocessing. mvp is an open‐source software that will be deposited at GitHub and CRAN to allow all users access to our software.

## Author contributions

GL and HN conceived and designed the project. BHJ and HBL acquired the data used for evaluating the performance of our software. GL and HN analyzed and interpreted the results from our software. GL, HBL, BHJ, and HN wrote the manuscript. GL implemented the software.

## Data accessibility

mvp is available at GitHub (https://github.com/GIST-CSBL/MVP).

## Supporting information


**Appendix S1.** Basic tutorial.
