# Supplementary material for: mvp – an open‐source preprocessor for cleaning duplicate records and missing values in mass spectrometry data
Source: FEBS Open Bio. 2017 Jun 19;7(7):1051–9. doi: 10.1002/2211-5463.12247 (PMC5494294; doi:10.1002/2211-5463.12247)
Supplement: Supplementary file 1 — Appendix S1. Basic tutorial. [file FEB4-7-1051-s001.pdf]

Supplementary Material

Basic Tutorial (Supplement 1)

Same content of this tutorial can be shown in main page of MVP(https://github.com/GIST-CSBL/MVP)

MVP

An implementation of Mass spectrometry Data Preprocessor

- MVP is an open-source software for preprocessing mass spectrometry data
- MVP is based on the R language and can be installed easily
- Users can set their own parameters to obtain preprocessed data for various situations.
- Duplicate record and missing value problems are alleviated by MVP
- Data preprocessed with MVP can improve the performance of statistical tests

---

Currently, you can install from GitHub with this command:

```
devtools::install_github("GIST-CSBL/MVP")
```

We checked installation from Windows, Ubuntu OS.

We will deposit MVP package to CRAN or Bioconductor.
After submission, we will announce the method to download CRAN or Bioconductor.

Basic usage (Tutorials)

In the package, we previde three example datasets that is reformated by first step. Users can access example dataset from this command.

```
load("data/ToF_Positive_Ion_Cardiovascular_Patient.rda")
load("data/ToF_Negative_Ion_Cardiovascular_Patient.rda")
load("data/Orbitrap_Drug_Treatment.rda")
```

Example data has form like this:

```
   `Primary ID Source` `Retention time (min)`      Mass `VIP[2]`     CType1
CType2    CType3     CType4      CType5
                 <chr>                      <dbl>     <dbl>    <chr>      <dbl>
<dbl>    <dbl>      <dbl>       <dbl>
1          Metabolite1                    11.3610 413.2665   75.6566         NA
801.22500 658.9790 829.51200 850.962000
2          Metabolite2                    11.3610 803.5420   52.9068         NA
385.30700 287.1710 461.28300 443.445000
3          Metabolite3                    11.3069 413.2666   50.4704 638.85000
1.94153 658.9790   1.80367   4.719440
4          Metabolite4                    11.3069 803.5420   34.9375 277.13400
```

```
NA 287.1710        NA         NA
5          Metabolite5              10.6577 379.2823  31.4945   5.82057
49.79090   2.2531  45.17050   7.397180
6          Metabolite6               9.7380 496.3401  26.8513 532.74500
622.35500 428.0600 553.41500 546.130000
7          Metabolite7               1.5148 100.0764  25.5866  78.38820
83.93800  67.1396 113.81900  73.692600
8          Metabolite8               9.7380 991.6699  23.5732 112.42800
184.42900  60.4640 165.55200 138.506000
9          Metabolite9              10.8200 524.3715  22.7365 193.15100
228.16600 147.8830 203.60400 190.575000
10        Metabolite10               3.4083 775.8630  19.5246        NA
NA        NA        NA   0.001488
# ... with 3,898 more rows, and 173 more variables: CType6 <dbl>, CType7
<dbl>, CType8 <dbl>, CType9 <dbl>
```

Data format reformating before applying MVP (1st step)

Each raw MS data has different format. For example, expression of missing value can be different (0, 1, NaN, …). Also, MVP need to know metadata (num of columns, intensity ratio columns …) Thus, before applying MVP, users should execute preparation method below.

```
reformated_data <- MVP::preprocess_input_data("Foo/MS_Data_Path", c(3, 2),
5:181, 0)
```

From example data format, we can see identifiers of MS data
The second and third column shows retention time and m/z ratio respectively
And from 5th to final column represent intensity signal of each patient.

First argument represent the path of input MS file
Second argument shows index of identifier (m/z, retention time …) column
Third argument should be set index of intensity signal column
Fourth argument is expression of missing value in raw data file

Another example is shown in below, for obtain this reformated data, users can input this command

```
       CompID    CompMW       MZ  Time Frames  MS2s  GoodIDs  Composition
Control1 Control2 Control3 Control4 Control5
        <chr>     <chr>     <dbl> <dbl>   <chr> <chr>    <chr>        <chr>
<dbl>    <dbl>    <dbl>    <dbl>    <dbl>
1    Compound1 110.1094 111.1167 16.45       4     0        0         C8H15
5451919  5859771  5607642  5596205  5622329
2    Compound2 113.0586 114.0659  0.98       8     0        0       C4H8ON3
8872545  7635595  8179503  6046026  5472458
3    Compound3  115.063 116.0703  0.99       6     1        0      C5H10O2N
18811215 22871758 19382179 20754701 25320232
4    Compound4 117.0577 118.0650  5.64       4    15        0          C8H8N
2497441  2360456  1714853  2196303  1682624
5    Compound5 117.0788 118.0860  1.00       8   194        0      C5H12O2N
25683704 25805455 25316523 22763418 25395905
6    Compound6 117.9717 118.9789  0.15       5   133        0          C5N2P
```

```
2567835  2341199  2539197  2489484  2644816
7   Compound7 117.9715 118.9788  0.41      1     0       0       C5N2P
2567519  2342544  2534912  2489798  2647408
8   Compound8 117.9716 118.9788  0.70      4     0       0       C5N2P
2681419  2422994  2662704  2599097  2765844
9   Compound9 117.9717 118.9790  6.82      2   933       0       C5N2P
30033529 31089689 30635252 32062554 33248868
10 Compound10 117.9716 118.9789  6.92      3   683       0       C5N2P
73795605 75341181 75415681 78072006 80433690
# ... with 3,224 more rows, and 115 more variables: Control6 <dbl>,
Control7 <dbl>, Control8 <dbl>, Control9 <dbl>
```

We can see that 3rd column is m/z ratio and 4th column is retention time
Therefore, this command can be executed for getting reformated data

```
reformated_data2 <- MVP::preprocess_input_data("Foo/MS_Data_Path", c(3,
4), 9:127, 0)
```

After preparing input data to reformated data, MVP can handle dirty data

MVP provide many user specified parameters.
To obtain dirty preprocessed data, execute this command

```
preprocessed_data_0.7 <-
MVP::apply_clique_method(ToF_Positive_Ion_Cardiovascular_Patient, c(3, 2),
c(0.001, 0.3), 5:181, 0.7)
[1] "Start Iteration 1"
  |======================================================================|
100%
[1] "# of records (Before): 3908 -> # of records (After): 3589"
[1] "Start Iteration 2"
  |======================================================================|
100%
[1] "# of records (Before): 3589 -> # of records (After): 3567"
[1] "Start Iteration 3"
  |======================================================================|
100%
[1] "# of records (Before): 3567 -> # of records (After): 3567"
```

First argument represent the reformated data from first step
Second argument shows index of identifier (m/z, retention time ...) column
Third argument is the similarity threshold of each identifier
Fourth argument should be set index of intensity signal column
Fifth argument is record similarity when merging duplicate candidate records

Users can specify parameters like this form

```
preprocessed_data_0.3 <- MVP::apply_clique_method(ToF_Positive_Ion_Cardiov

ascular_Patient, c(3, 2), c(0.001, 0.3), 5:181, 0.3)

preprocessed_data_different_threshold_of_identifier <- MVP::apply_clique_m
```

```
ethod(ToF_Positive_Ion_Cardiovascular_Patient, c(3, 2), c(0.004, 0.1), 5:1
81, 0.7)
```
